# Turning behavior links cognitive and motor function to safe mobility post-stroke: a cross-sectional mediation analysis

**DOI:** 10.3389/fneur.2026.1941016

**Published:** 2026-10-29

**Authors:** Anjali Tiwari, Stefan Delmas, Sharon N. Poisson, Neha Lodha

**Affiliations:** 1 Department of Health and Exercise Science, Colorado State University, Fort Collins, CO, United States; 2 Department of Neurology, School of Medicine, University of Colorado, Aurora, CO, United States

**Keywords:** ambulation, dual-task walking, mechanism, mediation, peak turning velocity, turning angle

## Abstract

**Introduction:**

Turning behavior is the fundamental component of safe mobility. Despite rehabilitation, nearly three-fourths of falls after stroke occur during turning maneuvers. Consequently, individuals with stroke experience persistent mobility limitations, highlighting turning as a clinically important yet comparatively under-assessed determinant of safe mobility. Because turning engages both cognitive and motor systems, it may serve as a key link between these functions and safe mobility. The current study examined whether turning is linked to safe mobility and mediates the associations between cognitive function, motor function, and safe mobility after stroke.

**Methods:**

One hundred and five stroke survivors participated in this study. Turning behavior was characterized by turn duration, turn angle, and peak turning velocity measured during dual-task walking. Safe mobility was assessed using Timed Up-and-Go (TUG) duration, Berg Balance Scale (BBS), gait speed, and gait variability score. We used confirmatory factor analysis (CFA) to compute latent scores for safe mobility. Cognitive function was assessed using an extensive neuropsychological battery. Motor function was evaluated using dorsiflexion and plantarflexion strength and motor accuracy on a goal-directed ankle task. We computed the composite score for turning behavior, cognitive, and motor functions. We conducted linear regression to examine the association between turning behavior and safe mobility, and mediation analyses to test whether turning behavior mediates the relationships of cognitive and motor function with safe mobility.

**Result:**

Poorer turning behavior was associated with compromised safe mobility (*R^2^* = 0.66). Mediation analysis revealed that turning mediated the relationship between cognitive function and safe mobility (indirect effect; *b* = 0.29, *SE* = 0.11, CI: 0.08 to 0.54). Similarly, turning mediated the relationship between motor function and safe mobility (indirect effect; *b* = 0.40, *SE* = 0.10, CI: 0.22 to 0.63).

**Conclusion:**

Turning behavior is a significant predictor of safe mobility post-stroke. Moreover, turning is a key pathway through which cognitive and motor impairments influence safe mobility after stroke. These findings suggest that incorporating turning-specific training may be an important consideration in gait rehabilitation post-stroke.

## Introduction

Turning behavior is the fundamental component of mobility, accounting for over 40% of daily movement in most adults (1, 2). For example, moving through constrained spaces at home, navigating an aisle in the grocery store, and walking a corner in the neighborhood all require the ability to make successful turns. Following stroke, turning becomes a disproportionately high-risk mobility behavior, with nearly three-fourths of falls occurring during turning maneuvers (3). Strikingly, post-stroke turning difficulties persist even when walking appears to be recovered (4–7). For example, individuals with stroke who ambulate well in a straight line continue to demonstrate poor turning behavior as evident in slower turns, requiring additional steps to complete a turn, and inefficient reorientation of the trunk and limbs while turning. These challenges in completing a turn are not merely biomechanical abnormalities but pose greater mobility limitations (8). For example, impaired turning heightens the fear of falling and reduces confidence during community ambulation, thus limiting the range of environments an individual with stroke can navigate safely. Despite this, stroke research and clinical rehabilitation efforts have predominantly focused on restoring gait performance primarily in a straight-line (9, 10). Therefore, the direct relationship between turning behavior and safe mobility post-stroke remains unclear (11, 12). Here, we define safe mobility as a multi-dimensional construct that includes balance and gait outcomes. This approach allows a comprehensive assessment of safe mobility rather than taking a piecemeal approach and measuring it with a single outcome in a specific domain (13).

Turning relies on the integration of cognitive and motor resources, both of which can be severely affected by stroke (4, 11, 14–18). Making a successful turn requires executive function, visuospatial processing, and attention to adapt the movement trajectories during directional change (11, 19). These cognitive requirements of turning far exceed those of steady, straight-line walking (20). Turning also imposes distinct demands on the motor system, requiring sufficient lower-limb strength, axial rotation, and optimal muscle tone that may be diminished following stroke (12, 21). Therefore, we posit that turning could serve as a “stress test,” exposing post-stroke mobility limitations that may otherwise remain unrecognized during standard clinical tests, focused on the relatively simpler task of straight-line walking. Although prior work in stroke literature has examined turning movements, to date, no study has examined the direct relationship between turning behavior and safe mobility, nor has any study identified whether turning serves as the link between cognitive-motor impairments and safe mobility after stroke.

The goal of this study is to determine the association between turning behavior and safe mobility after stroke and whether turning behavior mediates the relationship between cognitive and motor function and safe mobility in stroke survivors. We hypothesized that poor turning behavior would be associated with compromised safe mobility after stroke, and that turning behavior will mediate the relationship between post-stroke cognitive and motor impairments and compromised safe mobility. We characterized turning during a dual-task walking paradigm designed to mimic the cognitive load of real-world navigation. We operationalized safe mobility as a latent construct derived from multiple balance and gait variables using confirmatory factor analysis.

## Methods

### Participants

One hundred and five stroke survivors volunteered to participate in the study. Participants’ characteristics are presented in Table 1. The inclusion criteria for stroke survivors included: (1) age between 21 and 90 years; (2) diagnosis of stroke at least 6 months prior to enrollment; (3) ability to ambulate independently in a community setting. We excluded individuals with (1) any neurological disorder other than stroke, such as epilepsy, traumatic brain injury, Parkinson’s disease; (2) individuals with ataxia, or severe physical disability (i.e., bedridden or wheelchair bound) after stroke; (3) a history of alcohol and substance abuse; (4) an untreated sleep disorder (i.e., obstructive sleep apnea, chronic insomnia) and/or major psychiatric illness such as untreated severe depression, bipolar disorder, schizophrenia, and other mood disorders; or (5) uncorrected vision and auditory impairments. The Institutional Review Board of Colorado State University (CSU) approved the experimental procedures. Prior to study enrollment, all participants read and signed the approved informed consent form.

**Table 1:** Descriptive sample characteristics (*N* = 105).

Variables	Mean ± SD	Median [IQR]
Sociodemographic characteristics		
Age (years)	66.06 ± 13.09	69.70 [58.29–74.90]
Years of education (years),	15.80 ± 2.43	16 [14–18]
Women, *n*	45	-
Ethnicity, *n*		
Non-Hispanic White	98	-
African American	3	-
Asian	1	-
Hispanic	1	-
Other	2	-
Time since stroke (years)	4.30 ± 6.49	1.51 [3–5]
Type of stroke, *n*		
Ischemic	91	-
Hemorrhagic	12	-
Both	2	-
Side of the lesion, *n*		
Left	38	-
Right	46	-
n/a	9	-
Lesion location, *n*		
Supratentorial	70	-
Infratentorial	19	-
Mixed	8	-
n/a	8	-
Affected side, *n*		
Left	45	-
Right	44	-
Stroke severity (mRS; 0–6)		
0, %	17.1	-
1, %	42.9	-
2, %	29.5	-
3, %	7.6	-
4, %	2.9	-
Walking aid (*n*)	22	-
Comorbidity burden	4.21 ± 2.08	4 [3–5]
GDS	8.26 ± 6.38	7 [3–12]
PSQI	6.80 ± 3.31	7 [3–9]

*N* = 105. L, Left; R, Right; n/a, unknown; mRS, Modified Rankin Scale; GDS, Geriatric Depression Scale; PSQI, Pittsburgh Sleep Quality Index; “-”, not applicable; IQR, 25th – 75th percentile interquartile range.

### Experimental protocol and procedures

The experimental protocol consisted of a single session, lasting ~ 90 min, which included demographic and health questionnaires, assessments of turning behavior, safe mobility, and cognitive and motor function.

### Demographics and health questionnaires

We obtained participants’ age, sex, ethnicity, and education using a demographic questionnaire. We obtained stroke-related characteristics, including type of stroke, time since stroke, side of lesion, and affected extremity after stroke, from electronic health records. We assessed the physical disability after stroke using the Modified Rankin Scale (mRS), such that higher scores indicated greater disability (22). Further, we assessed health-related outcomes, including comorbidity burden, depression, and sleep quality. To assess comorbidity burden, we used a health history questionnaire and assigned a score of 1 to each comorbid condition; thus, higher scores indicated greater comorbidity burden. To assess depression, we used the 30-item Geriatric Depression Scale (GDS), with scores >11 indicating mild to severe depression (23). To assess sleep quality, we used the Pittsburgh Sleep Quality Index (PSQI), a 19-item self-reported questionnaire, where higher scores reflect poorer sleep quality (24).

### Turning behavior

Turning behavior was assessed using a dual-task walking paradigm designed to simulate real-world mobility. Participants performed overground walking for one minute and turned at a designated point, while simultaneously performing a Paced Auditory Serial Addition Test (PASAT) (25). During the PASAT, a single digit between 1 and 9 was presented via audio every 3 s, and participants verbally reported the running sum of the two most recently heard digits (23).

To quantify the turning behavior, we used six wireless inertial measurement units (IMUs; Opal, APDM Wearable Technologies, Portland, OR, USA), positioned bilaterally on the wrists and ankles, and unilaterally on the sternum and lumbar region. Each IMU sampled synchronously at 128 Hz. Raw sensor data were validated following each trial and stored for offline processing. Turning direction and amplitude were not experimentally constrained, allowing participants to turn naturally in their self-selected direction at the reference point. Mobility Lab software identified turns when the lumbar sensor recorded a horizontal rotation of at least 45°, with a duration between 0.5 and 10 s (26). We examined both the spatial and temporal turning measures, including turning angle, turning duration, and peak turning velocity. A smaller turning angle, turning velocity, and longer turning duration indicate poor turning behavior.

*Composite turning behavior score*. To compute the composite turning behavior, we calculated standardized z-scores for all turning measures relative to the pooled sample mean and standard deviation. Where necessary, scores were reversed so that higher z-scores reflected better turning performance. A composite turning behavior score was obtained by averaging the standardized values across all turning measures.

### Safe mobility

To obtain a comprehensive representation of safe and efficient real-world movement, safe mobility was assessed using multiple balance and gait-related outcomes (27–35). Specifically, we conducted the Timed Up and Go (TUG) test, the Berg Balance Scale (BBS), and the overground walking test. During the TUG test, we instructed participants to get up from a chair, walk a short distance, turn around, and return to the chair to sit (34, 36). We administered two TUG trials, and the trial with the shortest completion time was used for analysis. The BBS is a 14-item clinical assessment of static and dynamic balance (33). Longer TUG duration and a lower BBS score correspond to poorer safe mobility. Gait outcomes during overground walking included gait speed, stride length variability, and stride-time variability. Stride length and stride time variability were calculated as the cycle-to-cycle coefficient of variation (CV; std./mean*100) of the stride length and stride time, respectively. All gait outcomes were averaged across both extremities. Lower gait speed and higher stride length and stride time variability indicate worse overground mobility.

#### Safe mobility latent score

Here, we operationalized safe mobility as a single latent, multidimensional construct using balance and gait performance (27–34, 37). We computed a latent variable through confirmatory factor analysis (CFA) using five indicators, including TUG duration, Berg Balance Scale, gait speed, stride length, and stride time variability, to derive a robust and theoretically grounded latent representation of safe mobility. The CFA model was estimated using the Maximum Likelihood with Robust standard error (MLR) estimator to account for non-normality in the data. Individual-level latent factor scores for safe mobility were subsequently extracted using the regression method. The CFA model demonstrated favorable fit based on Comparative Fit Index (CFI) = 0.975, Tucker-Lewis Index (TLI) = 0.93, Standardized Root Mean Square Residual (SRMR) = 0.03, and Goodness of Fit (GIF) = 0.95 (38). However, RMSEA = 0.15, 90% CI: 0.08–0.23, exceeded the acceptable threshold fit. The inflated RMSEA is consistent with prior evidence that low degrees of freedom can substantially inflate the RMSEA and its confidence interval (39). All indicators showed factor loadings ranging from 0.72 to 0.90, indicating adequate representation of the latent safe mobility construct (Supplementary material, Table 3).

### Cognitive function

We measured cognitive function using an extensive neuropsychological battery that captured specific domains of attention, processing speed, memory, and executive function (40, 41). Specifically, the battery included the Useful Field of Vision (UFOV)- divided and selective attention subtest (42), Trail-making test (TMT) A and B (43), Digit Symbol Substitution Test (DSST) (44), Logical Memory Story - delayed recall, Hopkins Verbal Learning Test (HVLT) - delayed recall (45–47), Digit Span Test (DGT) forward and backward (48), and category fluency (49). Lower scores on DSST, Logical Memory, HVLT, and DGT forward and backward, and higher scores on UFOV subtests, and TMT A and B indicate poor cognitive function.

### Motor function

To measure motor function, we assessed the strength and motor accuracy of the ankle. Strength is a clinical indicator of stroke motor recovery. Motor accuracy reflects motor control and coordination (50, 51). Strength was measured as the maximum voluntary contraction (MVC) of dorsiflexion and plantarflexion for each ankle using an electronic manual muscle tester (Lafayette Manual Muscle tester, 01165, IEC 60601–1-2; 2007–03). Motor accuracy was measured during a goal-directed task across 30 trials for both ankles using previously published procedures (52, 53). Briefly, participants were required to reach a target force of 10 N in 180 ms with isometric dorsiflexion of the ankle. Accuracy was defined as the overall error computed as the hypotenuse of the force and time error (see prior publication for details) (54, 55).

### Composite scores

We computed 2 separate composite scores to represent cognitive and motor function. For each cognitive and motor measure, we computed standardized z-scores relative to the pooled sample mean and standard deviation. Where necessary, scores were reversed so that higher z-scores reflected better cognitive and motor performance. We subsequently computed composite cognitive and motor scores by averaging the standardized *z*-scores across all cognitive and motor measures, respectively.

### Statistical analysis

We used descriptive statistics to summarize the participants’ characteristics. Here, we expressed the continuous variables using mean and standard deviation, and the categorical variables using frequencies or percentages. Before the analysis, the distribution of continuous variables was assessed for normality.

To determine the relationship between turning behavior and safe mobility, we conducted linear regression analysis with turning behavior as the independent variable and safe mobility as the dependent variable. Unstandardized (b) and standardized regression coefficients (β), standard errors, and *p*-values were reported to quantify the strength and direction of associations. To determine whether turning behavior serves as a mediator between cognitive and motor function and safe mobility, two separate cross-sectional mediation analyses were conducted. In the first model (*Model 1*), we included cognitive function as the independent variable, turning behavior as a mediator, and safe mobility as a dependent variable. For the second model (*Model 2*), we included motor function as an independent variable, turning behavior as a mediator, and safe mobility as a dependent variable. To account for the potential confounding effects of demographic and clinical characteristics on these indirect-effect models, we conducted covariate-adjusted sensitivity analyses for both models. Covariates that demonstrated significant associations with the independent variable, mediator, and dependent variable were included in the analysis, including age, sex, education, and stroke severity (mRS), with correlation coefficients ranging from |*r*| = 0.21–0.53. Standardized and unstandardized coefficients, standard errors, and confidence intervals for all the path coefficients, including direct, indirect effects, and total effects, were estimated using bias-corrected bootstrap resampling with 5,000 bootstrap samples. All model parameters with 95% confidence intervals are reported. All statistical analyses were conducted using RStudio (version 2024.0.4.2) and SPSS 29.0 (IBM, Armonk, NY), with the alpha level set at 0.05.

*Missing values:* Missing data were minimal across all the measures (<5%). The missing values were handled using multiple imputation for continuous variables and mode substitution for categorical variables.

*Sample size determination:* To achieve 80% power at an alpha level of 0.05 and a moderate effect size, 87 participants were needed to address aim 1. For aim 2, an *a priori* power analysis was conducted using Fritz and MacKinnon’s (2007) guidelines for mediation analysis (56). Based on these guidelines, a sample size of 71 was required to detect a mediated effect of medium size at 80% power and alpha = 0.05 using the bias-corrected bootstrap method.

## Results

### Participants demographics

The participants’ characteristics are presented in Table 1. The stroke participants had a mean age of 66 years, were highly educated with an average of 16 years of education, and 43% of the participants were women. Most participants were non-Hispanic white (>93%), and the majority experienced ischemic (>86%) and supratentorial (>66%) stroke and had mild to moderate disability (mRS = 0–2; 89.5%). The mean GDS score was 8, and PSQI was 7, demonstrating poor sleep quality among stroke survivors. The performance on cognitive, motor, turning, and safe mobility variables is shown in Table 2.

**Table 2:** Descriptive statistics of turning and safe mobility, cognitive, and motor variables.

Variables	Mean ± SD	Median [IQR]
Turning behavior		
Turning angle (^o^)	175.51 ± 20.23	180.10 [174.73–185.32]
Turning duration (s)	2.72 ± 0.51	2.65 [2.39–2.98]
Peak Turning Velocity (^o^/s)	138.11 ± 32.69	138.51 [114.73–158.08]
Safe mobility		
Time Up and Go test (s)	12.77 ± 4.77	11.40 [10.20–13.70]
Berg Balance Scale (/56)	49.58 ± 7.45	53 [46–55]
Gait speed (m/s)	0.91 ± 0.24	0.95 [0.79–1.08]
Stride Length variability (%)	4.53 ± 2.52	3.77 [2.83–5.22]
Stride time variability (%)	3.42 ± 1.88	2.78 [2.10–3.99]
Cognitive function		
Divided attention (ms)	162.92 ± 151.58	113 [28.25–240]
Selective attention (ms)	146.66 ± 141.53	233 [141.50–350.75]
Trail-making A (s)	45.52 ± 43.97	35.05 [25.06–44.54]
DSST (*n*)	52.53 ± 18.99	52.5 [42–65]
DGT – Forward (*n*)	9.56 ± 2.15	9 [8–11]
Logical memory-Delayed (*n*)	9.30 ± 3.24	10 [7–12]
HVLT- Delayed (*n*)	7.02 ± 3.52	8 [5–10]
Trail making B (s)	109.56 ± 72.06	84.55 [62.81–11]
Category fluency	19.73 ± 6.62	19 [17–23]
DGT -Backward (*n*)	7.64 ± 2.09	8 [6–9]
Motor function		
Dorsiflexion strength -affected (N)	142.70 ± 43.48	142.55 [121.02–169.17]
Dorsiflexion strength -unaffected (N)	150.57 ± 40.02	149.50 [126.92–176.50]
Plantarflexion strength- affected (N)	163.45 ± 54.60	153.45 [121.42–203.07]
Plantarflexion strength- unaffected (N)	170.66 ± 53.94	166.35 [131.52–202.47]
Motor accuracy – affected (%)	124 ± 11.56	75.70 [53.22–127.84]
Motor accuracy – unaffected (%)	107 ± 94.32	79.77 [48.52–123.28]

HVLT, Hopkins Verbal Learning Test; DSST, Digit Symbol Substitution Test; DGT, Digit Span Test; N, Newton; IQR, 25th – 75th percentile interquartile range.

### Relationship between turning behavior and safe mobility

The linear regression analysis revealed that turning behavior significantly predicted safe mobility, *F* (1,103) = 203.86, *p* < 0.001, accounting for 66% of the variance (*R^2^* = 0.66), which represents a large effect size based on Cohen’s guidelines (57). Better turning behavior was positively associated with safe mobility (b = 1.07, SE = 0.07, CI: 0.92 to 1.21) in stroke survivors (Figure 1; Table 3).

**Figure 1:**
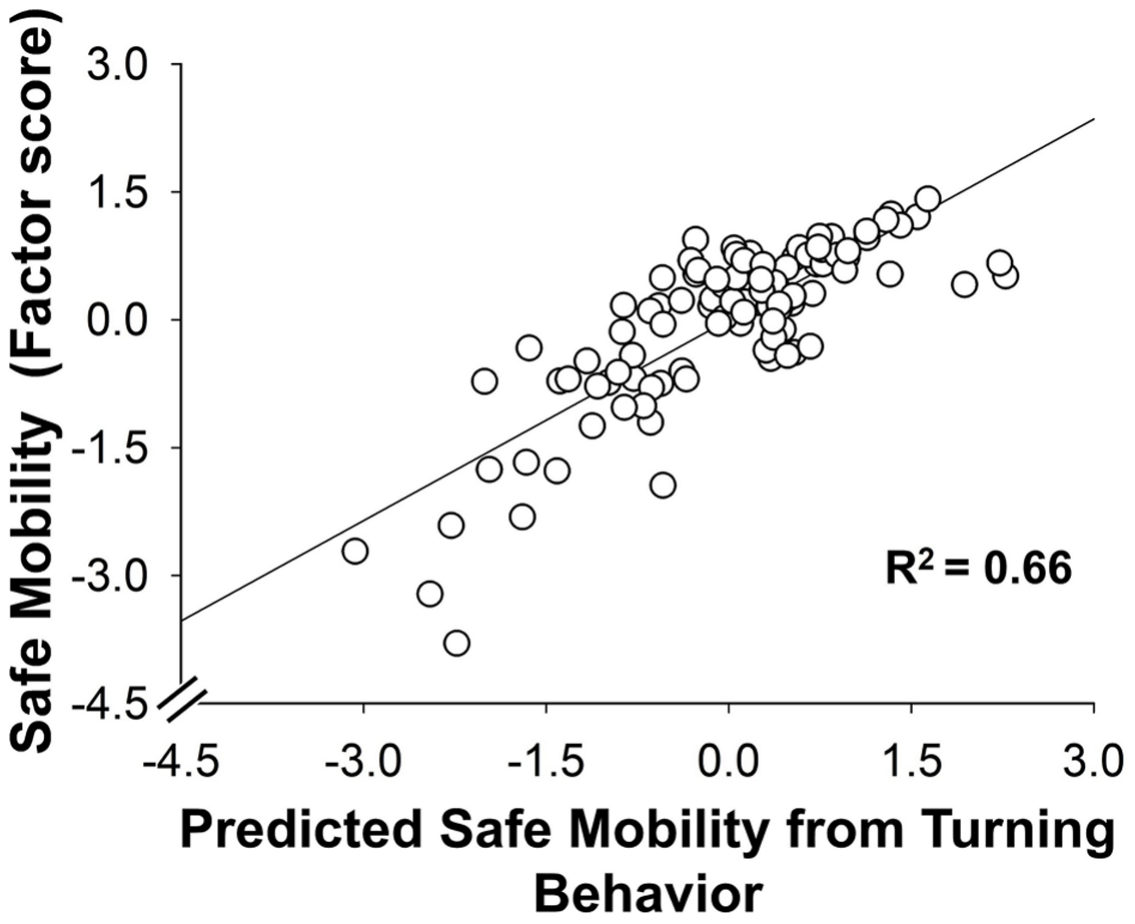
Turning behavior predicts the safe mobility construct. Linear regression was used to examine the association between turning behavior and safe mobility. The *x*-axis represents safe mobility scores predicted from the turning behavior composite, and the *y*-axis represents the observed safe mobility factor scores. The model explains 66% of the variance (*R*^2^ = 0.66) in observed safe mobility explained by turning behavior.

**Table 3:** Linear regression and mediation model.

Linear regression	B	SE	β	95% CI		*p*
				LL	UL	
Turning behavior→ Safe mobility	1.07	0.07	0.81	0.92	1.21	< 0.001
Model 1: Mediation Model (Cognitive function)						
Regression paths						
a: Cognition → Turning	0.29	0.11	0.28	0.09	0.49	< 0.01
b: Turning → Safe mobility	0.98	0.07	0.74	0.83	1.12	< 0.001
Effects						
Indirect effect (a x b)	0.29	0.11	0.20	0.08	0.54	< 0.01
Direct effect (c’)	0.32	0.07	0.23	0.17	0.48	< 0.001
Total effect	0.52	0.12	0.44	0.37	0.86	< 0.001
Model 2: Mediation Model (Motor function)						
Regression paths						
a: Motor → Turning	0.44	0.09	0.41	0.25	0.63	< 0.001
b: Turning → Safe mobility	0.91	0.07	0.70	0.77	1.06	< 0.001
Effects						
Indirect effect (a x b)	0.40	0.10	0.29	0.22	0.63	< 0.001
Direct effect (c’)	0.38	0.07	0.27	0.22	0.54	< 0.001
Total effect	0.79	0.11	0.56	0.56	1.01	< 0.001

B, unstandardized coefficient; SE, standard error; β, standardized coefficient; 95% CI, confidence interval (95% bootstrapped); a, independent variable to mediator; b, mediator to outcome variable; c^`^, direct effect.

### Cross-sectional mediation analysis of turning behavior as a mediator of cognitive function and safe mobility

Mediation analysis for model 1 revealed that turning behavior significantly mediated the association between cognitive function and safe mobility (indirect effect (a*b); *b* = 0.29, *SE* = 0.11, CI: 0.08 to 0.54). The association between cognitive function and safe mobility was attenuated but remained significant when controlling for turning behavior (direct effect (c’); *b* = 0.32, *SE* = 0.07, CI: 0.17 to 0.48), indicating partial mediation. Overall, cognitive function was significantly associated with safe mobility (total effect (c); *b* = 0.52, *SE* = 0.12, CI: 0.37 to 0.86) (Table 3; Figure 2A).

**Figure 2:**
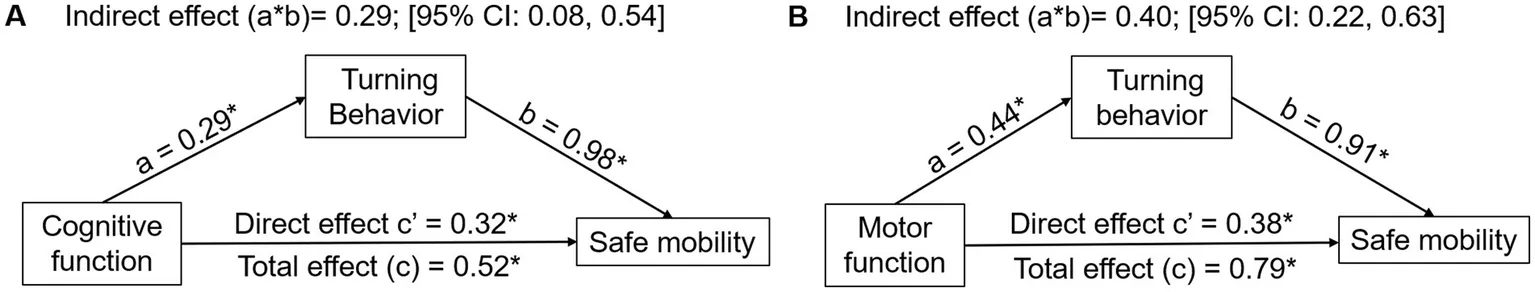
Mediation model. **(A)** Turning behavior mediated the relationship between cognitive function and safe mobility after stroke. **(B)** Turning behavior mediated the relationship between motor function and safe mobility after stroke.

### Cross-sectional mediation analysis of turning behavior as a mediator of motor function and safe mobility

Mediation analysis for model 2 revealed that turning behavior significantly mediated the association between motor function and safe mobility (indirect effect (a*b); *b* = 0.40, *SE* = 0.10, CI: 0.22 to 0.63). The association between motor function and safe mobility was attenuated but remained significant when controlling for the turning behavior (direct effect (c’); *b* = 0.38, *SE* = 0.07, CI: 0.22 to 0.54), indicating partial mediation. Overall, motor function was significantly associated with safe mobility (total effect (c); *b* = 0.79, *SE* = 0.11, CI: 0.56 to 1.01) (Table 3; Figure 2B).

### Covariate-adjusted sensitivity analyses

To evaluate the robustness of the observed indirect effects, we conducted covariate-adjusted sensitivity mediation models, controlling for age, sex, education, and stroke severity (mRS). The linear regression model revealed that turning behavior significantly predicted safe mobility, *F* (5,99) = 60.74, *p* < 0.001, accounting for 75% of the variance (*R^2^* = 0.75). In Model 1, the indirect effect of cognitive function on safe mobility through turning behavior remained significant after covariate adjustment (indirect effect (a*b); *b* = 0.19, *SE* = 0.09, CI: 0.01 to 0.39). In Model 2, the indirect effect of motor function on safe mobility through turning behavior remained significant after covariate adjustment (indirect effect (a*b); *b* = 0.26, *SE* = 0.06, CI: 0.15 to 0.40) (please see details in the Supplementary material, Table 1).

## Discussion

The current study examined whether turning behavior is directly associated with safe mobility and whether it serves as a potential pathway linking cognitive and motor function to safe mobility outcomes following stroke. Two main findings emerged. First, poor turning behavior is associated with compromised safe mobility in stroke survivors. Second, turning behavior mediates the association between cognitive and motor function and safe mobility. To our knowledge, the current study is the first to show that turning behavior plausibly links cognitive and motor function to safe mobility outcomes post-stroke. Conceptually, our findings suggest that turning behavior may represent an integrated functional expression of cognitive and motor capacities that contribute to safe mobility after stroke. Clinically, our results highlight that turning behavior links to safe mobility, suggesting that incorporating turning-specific training may be an important consideration in gait rehabilitation after stroke.

### Turning behavior: potential pathway linking cognitive and motor impairment to safe mobility after stroke

Turning behavior might represent an important potential functional pathway linking cognitive impairment to safe mobility after stroke. This link may potentially be explained by the fact that turning maneuvers require higher-order cognitive processing to continuously update spatial orientation and adapt movement trajectories within a dynamically changing environment. As a result, turning behavior likely places greater demands on executive, attention, and visuospatial systems than straight-line walking. Supporting this mechanism, prior work has demonstrated that cognitive neural systems are comparatively more actively engaged during turning navigation than simple navigation in a straight-line. For example, premotor cortical lesions are associated with impaired turning behavior, highlighting the role of motor planning and increased cognitive load in directional changes during walking (58). Similarly, increased attentional loading during dual-task walking delayed axial segment reorientation as compared with straight-line walking (59). Turning mobility has also been shown to be associated with visuospatial ability and language after stroke, with balance and lower-limb strength identified as mediators (11). The current study extends this literature by examining turning behavior as a possible mediator linking cognitive and motor function to safe mobility outcomes after stroke. Taken together, these findings suggest that post-stroke cognitive impairment may disrupt turning behavior, thereby compromising an individual’s ability to safely ambulate in the community.

Another notable finding is that turning may serve as a critical functional pathway through which motor impairments influence safe mobility after stroke. This relationship may be explained by the distinct neuromuscular and biomechanical demands required during turning (7, 8, 60, 61). Stroke-related damage to the motor pathways reduces neuromuscular activation, leading to reduced muscle strength, altered muscle tone, impaired rotational torques, and inter-limb coordination (62–64). For example, reduced neuromuscular modulation in the paretic limb and lower-limb excursion adversely affect turning performance (18). Biomechanically, impaired postural adaptation leads to longer turn duration and slower turning velocity, contributing to increased instability and fall risk during ambulation after stroke (20, 62, 65, 66). Therefore, impaired lower-limb coordination and balance post-stroke may compromise the rapid adjustments required for efficient turning behavior and contribute to gait asymmetry and unsafe mobility (16, 67). Additionally, although stroke survivors often rely on compensatory strategies such as pelvic hiking and circumduction during walking, these compensatory strategies may not work during turning due to higher biomechanical and postural demands (66). Overall, these findings suggest that turning behavior, as a motorically demanding task, may serve as a potential link through which impaired motor function influences safe mobility after stroke.

In summary, our findings suggest that turning represents a convergence point through which both cognitive and motor impairments could be translated into real-world mobility limitations after stroke. As a cognitively and motorically demanding task, turning may function as a potential link between these impairments and safe mobility, thus capturing deficits that are not fully reflected in straight-line gait assessments.

### Clinical implication

Despite significant post-stroke rehabilitation efforts, mobility limitations remain a common and persistent challenge in stroke recovery. Our findings suggest that turning behavior may provide unique insights into an individual’s capacity for safe navigation and may be particularly informative for identifying those at risk for unsafe mobility after stroke. Along these lines, a prior study showed that slower turning duration and increased number of steps predicted future falls among stroke survivors (68). Further, turning-specific training has been shown to improve gait asymmetry, balance control, and coordination among chronic stroke survivors (69). Therefore, turning behavior may serve as a clinically meaningful functional target to enhance safe mobility in stroke survivors. Overall, integrating assessments and rehabilitation of turning into routine clinical practice may enhance safe mobility following stroke.

### Limitation and future considerations

This study has some limitations that should be considered when interpreting the findings. First, the study sample was predominantly high-functioning individuals with stroke (mRS = 1.36), highly educated and non-Hispanic White, which may limit the generalizability of the results to more impaired and demographically diverse stroke populations. Second, the cross-sectional design of the current study limits the ability to draw causal inferences regarding the observed mediation effects. Although the findings suggest that turning performance may serve as a potential pathway linking cognitive and motor function to safe mobility, longitudinal studies are needed to establish the temporal and directional nature of the relationships. Third, turning performance could be influenced by sensory processes, including proprioceptive and somatosensory function, which were not assessed in the present study. Future research should incorporate comprehensive sensory evaluations to better understand the contribution of sensory impairments to turning performance and mobility outcomes after stroke. Further, turning behavior was assessed under a dual-task condition using the PASAT; however, PASAT performance alone was not measured. Therefore, the potential contribution of cognitive-task performance and dual-task prioritization to turning behavior could not be determined. Future studies should consider the dual-task cost to evaluate the influence of cognitive load on turning behavior. Further, the current study did not account for the potential influence of gait speed, turning amplitude, or turning direction on turning (70). Finally, although safe mobility was assessed using validated gait and balance measures, direct assessment of future falls was not included. Future studies should incorporate prospective fall monitoring to further validate the current findings.

## Conclusion

Turning emerges as a unified functional marker of cognitive and motor capacity, capturing the convergent demands that underlie safe mobility after stroke. Specifically, turning behavior emerged as a possible key link through which cognitive and motor impairments contribute to compromised safe mobility after stroke. Clinically, including turning-specific assessment and gait rehabilitation may be an important consideration for promoting safe mobility after stroke.

## Data Availability

The raw data supporting the conclusions of this article will be made available by the authors, without undue reservation.
