# Supplementary material for: Turning behavior links cognitive and motor function to safe mobility post-stroke: a cross-sectional mediation analysis
Source: Front Neurol. 2026 Oct 29;17:1941016. doi: 10.3389/fneur.2026.1941016 (PMC13754663; doi:10.3389/fneur.2026.1941016)
Supplement: Supplementary file 1 [file Table_1.docx]

**Supplementary file**

**Table 1.** Sensitivity analysis -multiple linear regression and mediation model with covariates

| **Linear Regression** | **B** | **SE** | **β** | **95% CI** | | **P** |
| --- | --- | --- | --- | --- | --- | --- |
|  |  |  |  | LL | UL |  |
| Turning behavior→ Safe mobility (**R^2^ = 0.75**) | 0.87 | 0.07 | 0.66 | 0.72 | 1.02 | < 0.001 |
| **Model 1**: Mediation Model (Cognitive function) | | | | | | |
| *Regression paths* |  |  |  |  |  |  |
| a: Cognition → Turning | 0.34 | 0.10 | 0.32 | 0.13 | 0.54 | 0.001 |
| b: Turning → Safe mobility | 0.78 | 0.07 | 0.59 | 0.63 | 0.92 | < 0.001 |
| *Effects* | | | | | | |
| Indirect effect (a x b) | 0.19 | 0.09 | 0.14 | 0.01 | 0.39 | < 0.01 |
| Direct effect (c**’**) | 0.28 | 0.06 | 0.20 | 0.15 | 0.42 | < 0.001 |
| Total effect | 0.48 | 0.10 | 0.35 | 0.27 | 0.70 | < 0.001 |
| **Model 2:** Mediation Model (Motor function) | | | | | | |
| *Regression paths* |  |  |  |  |  |  |
| a: Motor → Turning | 0.32 | 0.10 | 0.30 | 0.12 | 0.52 | < 0.001 |
| b: Turning → Safe mobility | 0.78 | 0.07 | 0.59 | 0.63 | 0.92 | < 0.001 |
| *Effects* | | | | | | |
| Indirect effect (a x b) | 0.26 | 0.06 | 0.19 | 0.15 | 0.40 | < 0.001 |
| Direct effect (c**’**) | 0.31 | 0.08 | 0.22 | 0.15 | 0.47 | < 0.001 |
| Total effect | 0.58 | 0.11 | 0.41 | 0.36 | 0.80 | < 0.001 |

*Note.* B = unstandardized coefficient; SE = standard error; β = standardized coefficient; 95 % CI = confidence interval (95% bootstrapped); a= independent variable to mediator; b = mediator to outcome variable; c**^`^**= direct effect.

**Table 2.** Sensitivity analysis excluding the Time Up Go test (TUG) from the CFA

*Fit indices: Chi-square = 0.59; CFI =1; TLI=1; RMSEA=-.00 [0.000, 0.246]; SRMR=0.006; GFI=1; Standardized factor loadings for the Safe Mobility factor ranged from 0.718 to 0.912; degrees of freedom =1.*

| **Linear Regression** | **B** | **SE** | **β** | **95% CI** | | **P** |
| --- | --- | --- | --- | --- | --- | --- |
|  |  |  |  | LL | UL |  |
| Turning behavior→ Safe mobility (R^2^ = 0.65) | 1.04 | 0.07 | 0.80 | 0.89 | 1.19 | < 0.001 |
| **Model 1**: Mediation Model (Cognitive function) | | | | | | |
| *Regression paths* |  |  |  |  |  |  |
| a: Cognition → Turning | 0.29 | 0.10 | 0.28 | 0.09 | 0.49 | < 0.001 |
| b: Turning → Safe mobility | 0.94 | 0.07 | 0.73 | 0.80 | 1.09 | < 0.001 |
| *Effects* | | | | | | |
| Indirect effect (a x b) | 0.28 | 0.10 | 0.20 | 0.08 | 0.51 | < 0.01 |
| Direct effect (c**’**) | 0.34 | 0.07 | 0.25 | 0.19 | 0.49 | < 0.001 |
| Total effect | 0.62 | 0.11 | 0.46 | 0.39 | 0.86 | < 0.001 |
| **Model 2:** Mediation Model (Motor function) | | | | | | |
| *Regression paths* |  |  |  |  |  |  |
| a: Motor → Turning | 0.44 | 0.09 | 0.41 | 0.25 | 0.63 | < 0.001 |
| b: Turning → Safe mobility | 0.81 | 0.07 | 0.69 | 0.67 | 0.94 | < 0.001 |
| *Effects* | | | | | | |
| Indirect effect (a x b) | 0.36 | 0.07 | 0.28 | 0.21 | 0.55 | < 0.001 |
| Direct effect (c**’**) | 0.34 | 0.07 | 0.27 | 0.19 | 0.48 | < 0.001 |
| Total effect | 0.70 | 0.10 | 0.55 | 0.49 | 0.90 | < 0.001 |

*Note.* B = unstandardized coefficient; SE = standard error; β = standardized coefficient; 95 % CI = confidence interval (95% bootstrapped); a= independent variable to mediator; b = mediator to outcome variable; c**^`^**= direct effect.

**Table 3.** Confirmatory factor analysis: model fit indices:

(Factor determinacy score =0.94; degrees of freedom =4)

*CFA Model Fit Indices and factor loadings for the Safe Mobility Latent factor.*

| **CFA Model** | **χ²** | **CFI** | | **TLI** | | **RMSEA** | **SRMR** | **GIF** |
| --- | --- | --- | --- | --- | --- | --- | --- | --- |
| Safe Mobility | 14.18 | 0.97 | | 0.93 | | 0.15 | 0.03 | 0.95 |
| *Acceptable fit thresholds* | *p > .05* | *≥ .95* | | *≥ .95* | | *≤ .06* | *≤ .08* | *>0.90* |
| **Indicators**  *(Safe Mobility Factor)* | **B** | | **SE** | | **β** | | **p** | **Residuals** |
| Gait Speed (ms) | 0.89 | | 0.07 | | *0.90* | | *0.00* | *0.18* |
| Stride Length CV (%) | 0.80 | | 0.11 | | *0.81* | | *0.00* | *0.33* |
| Stride Time CV (%) | 0.71 | | 0.10 | | *0.72* | | *0.00* | *0.47* |
| TUG (sec) | 0.88 | | 0.17 | | *0.88* | | *0.00* | *0.21* |
| BBS (/56) | 0.77 | | 0.14 | | *0.77* | | *0.00* | *0.39* |

*Note.* CFA = Confirmatory Factor analysis; CFI = Comparative Fit Index; TLI = Tucker-Lewis Index;

RMSEA = Root Mean; Square Error of Approximation (robust); SRMR = Standardized Root Mean

Square; Residual; GIF = Goodness of Fit; ; B = unstandardized loading; SE = standard error; β =

standardized loading.
